# Mental health: “it is a subject where most pharmacists [or pharmacy] students have no more knowledge than the general public”

**DOI:** 10.1186/s40545-022-00489-x

**Published:** 2023-01-24

**Authors:** H. C. Gorton, J. Strawbridge, H. Macfarlane

**Affiliations:** 1 Department of Pharmacy, School of Applied Sciences, University of Huddersfield, Queensgate, Huddersfield, HD1 3DH UK; 2 School of Pharmacy and Biomolecular Sciences, Royal College of Surgeons in Ireland, Dublin, Ireland; 3 School of Pharmacy, College of Health and Life Sciences, Aston University, Birmingham, UK

**Keywords:** Mental health, MPharm, Undergraduate, Pharmacy curriculum, Pharmacy education

## Abstract

**Background:**

Mental health is a global health priority, and pharmacists have a valuable role in improving outcomes in all sectors of practice. This study sought to explore pharmacy students’ views on teaching and learning of mental health and future practice.

**Methods:**

An anonymous online questionnaire was distributed to pharmacy students in the UK and Ireland in February 2020 via the Qualtrics™ platform and 232 students responded. The questionnaire was originally intended to explore the provision of Mental Health First Aid (MHFA) teaching and the quantitative analysis has been previously reported. Students were invited to comment on their views about MHFA. The open-ended question: ‘Do you have any other comments about mental health teaching and learning in the MPharm degree?’ was also included. The rich free-text data were analysed, and themes identified.

**Results:**

Three major themes were identified: (i) Mental Health is important; (ii) Pharmacist roles and (iii) So, Teach me. A fourth theme, Stigma, crosscut all the themes.

**Conclusions:**

Pharmacy students appreciate the importance of mental health care. The majority recognise the role of the pharmacist in providing person-centred care and the potential to enhance this role. Students are keen to learn more, and acquire the confidence and skills to contribute in the future. They would like an integrated approach and have more opportunities to learn from patients. Addressing stigma is an important consideration for educators.

## Background

The improvement of mental health globally remains a key commitment of the World Health Organization (WHO), exemplified by the 10-year extension of their Mental Health Action Plan, to 2030 [1]. In the UK, four sets of mental health research goals have been identified [2] in order to focus research and drive improvement. Indeed, there is a need to understand how the COVID-19 pandemic and recovery might influence people’s mental health [3]. There is a clear emphasis on a multidisciplinary approach to mental health care, extending across sectors and within healthcare, including pharmacists and their teams.

Notwithstanding the recognised value of specialist mental health pharmacists, [4] in the UK, there is some increasing emphasis on the role that all pharmacists have in helping people with their mental health. In 2018, The Royal Pharmaceutical Society published their position statement [5] on the integral role of pharmacy in supporting people with their mental health and in 2020, Health Education England published their framework of mental health core competencies [6] that all pharmacy professionals are expected to meet, regardless of sector of practice.

In their recent review, El-den et al. [7] reconfirmed that pharmacists have a valuable role in mental health care, but more work is needed to evidence both clinical impact on patient’s care and economic outcomes such as cost-effectiveness from a health service perspective. Selected examples of community pharmacy based mental health services, in different stages of development worldwide include: the government-funded Bloom Program in Nova Scotia, Canada, [8–10] PharMIbridge trial in Australia [11] and the feasibility pilot of AMPLIPHY in Greater Manchester, UK [12]. Pharmacy staff have identified their role in suicide prevention and called for training provision [13]. In 2021, incentives for community pharmacies in England were provided if their patient-facing staff completed Zero Suicide Alliance training [14]. To support these enhanced roles, pharmacy education in mental health must grow [7].

Rutter et al. [15] reported on the mental health components of UK undergraduate pharmacy degrees in 2013. They revealed a theoretical focus, with little exposure to people with lived experience nor on effective communication. A similar picture was reported recently, with focus retained on therapeutics and neurobiology rather than problem-solving and person-centred care [16]. In this study, the role of Mental Health First Aid (MHFA) training for undergraduate pharmacy students was specifically explored. Just ten percent of respondents had undertaken this training, and the majority of those who had not would welcome the opportunity. The incorporation of MHFA into the pharmacy degree has been most widely explored in Australia [17, 18] and Ireland, [19] offering a promising route forward.

The aim of this study is to explore pharmacy students’ reflections on teaching and learning of mental health within pharmacy undergraduate degrees, with their perceived applications to practice.

## Methods

The methods used for this study have previously been described in full, when reporting the quantitative findings [16]. In brief, an anonymous, online questionnaire, hosted via Qualtrics™, was distributed to pharmacy students in 2 weeks of February 2020, in the UK and Ireland. 232 students responded from 18 universities. The questionnaire was shared via personal and professional networks, including through contact with colleagues across universities for cascade, and via social media networks including Twitter.

### Questionnaire

The original aim of the questionnaire [16] was to establish the attitudes and experiences of Mental Health First Aid (MHFA) Training. It contained quantitative questions, which have been reported on, and questions that enabled qualitative responses. This is the focus of this paper. To explore the broader context, open-ended space for students to comment on their responses to questions about their desire to participate in MHFA was included. Additionally, the following open-ended question was included: ‘Do you have any other comments about mental health teaching and learning in the MPharm degree?’. The large amount of rich, qualitative comments provided warranted dedicated interrogation.

### Ethical approval

This study was approved by the University of Huddersfield School of Applied Sciences Research, Integrity, and Ethics Committee (SAS-SREIC 11.02.20-1).

### Data analysis

The process of thematic analysis outline by Braun and Clark was followed [20]. Two authors (HCG, HM) independently read and re-read open comments to develop understanding of the main themes discussed when students reflected on mental health teaching, their own learning, and the anticipated value of this in professional practice. To ensure rigour, the authors initially undertook initial complete coding of all qualitative data. Themes were developed separately and then those which best described the data were agreed. A third author, JS, reviewed the coding, agreed the proposed themes, and revised subthemes. All authors agreed on the final themes and subthemes. The quotes are labelled with university and student year group. To maintain anonymity, students in years 4 and 5 are labelled as final year because labelling year 5 would likely reveal the university.

## Results

Pharmacy students clearly described mental health as being important to everyone. They described not only their perception of the relevance of mental health to their future roles as pharmacists, but also a sense that they had received insufficient education to fulfil this role. As such, three major themes were identified: (i) Mental Health is important; (ii) Pharmacist roles and (iii) So, Teach me. A fourth theme, Stigma, crosscut these themes.

### Mental health is important

Pharmacy students overwhelmingly considered mental health to be important but under-recognised and overlooked, particularly in comparison to physical illnesses. Some described their own experience of mental health problems and related this to the importance of making improvements to education and awareness more generally.

#### Recognising mental illness

Pharmacy students identified that pharmacists in all sectors should have an awareness of mental health signs, potential progression of symptoms and ability to identify and support people in mental health crisis such as suicidal thoughts or plans.

*“Mental health is a growing problem in society and as a healthcare professional we must be adept at identifying and aiding in mental crises.” (University 2, year 3).*

#### Mental health should have parity with physical health

Students perceived mental health to be overlooked, particularly compared to physical health, and linked this with a need to enhance awareness and improve outcomes for people living with mental illness.

*“I believe mental health is extremely important and often overlooked. Caring for someone’s mental health can save their life which is equally as important as the medicine they take.” (University 23, year 3).*

Pharmacy students suggested that there is less treatment and poorer service accessibility for mental health problems than for physical health problems.

*“I believe many people treat mental health patients differently from other patients. But I believe its our duty as HCPs to help someone in need just like we will do with someone with any other health condition.” (University 25, year 3).*

There was acknowledgement that it is pharmacists’ responsibility to drive equity, as much as other health care professionals.

*“Mental health is just as important as physical health and for some time the issue of mental health has not been focused on enough by the NHS. It is important that pharmacists know how to deal with patients that may come to them as a form of reaching out—it’s not just the responsibility of GPs.” (University 19, year 3).*

Comparisons were made with physical emergencies, such as sepsis and cardiac arrest, where concentrated training efforts were made based on significant events. Similarly, students compared lack of mental health first aid to physical health first aid, which has wider uptake. This comparison is not emphasised because these comments were likely biased by the focus of the questionnaire (MHFA) from which these data were drawn from.

*“It’s like sepsis, people were dying at a much faster rate than previously seen so the NHS trained their staff to recognise the first stages and put into place the necessary steps to save a life. For me, this needs to be done for mental health as it is just as deadly but not as visible.” (University 25, final year).*

Some students described that physical and mental health are intertwined, and not mutually exclusive. Therefore, a focus on mental health could result in improvements to the physical health agenda.

*“Mental health should not be seen as any less important than physical health, especially given the strong association between the two.” (University 5, final year).*

#### Personal experience of mental health problems

Some students discussed their experience of mental health problems in their personal life as a motivator to improve their practice, and that of their peers.

*“I have unfortunately received poor mental health care, and do not wish patients to have the same experience.” (University 5, final year).*

Students outlined the importance of promoting mental health and wellness. This included how curricula supports their own mental health, in the context as their role as students and aspiring pharmacists. Some suggested more focus on this.

*“I think there should be more of a focus on how to keep your mental health healthy as opposed to focusing on it in the context of sickness.” (University 18, final year).*

*“Also it would be good if it was embedded into the syllabus each year as we are all students so we can all help to look out for one another.” (University 25, year 1).*

Conversely, another felt that there was too much emphasis on mental health from the personal viewpoint.

*“Our mental health as pharmacists seems to be discussed more regularly than the patients we will see on a day to day basis.” (University 19, final year).*

### Pharmacist role

Respondents were clear about the value of the role of pharmacists in mental health care, with some going further to identify aspects which warrant improvement.

#### Providing person-centred care

The importance of a patient-centred approach in practice was identified and students linked this with the amount pharmacists engage with mental health.

*“Patients should feel welcomed by pharmacists so that they can openly talk about their health concerns. Thus, communication skills are critical.” (University 18, final year).*

*“It is becoming a larger area of care within the general population and Health Care Professionals need to be taught on what's best for patients.” (University 22, year 3).*

Where respondents have used ‘pharmacists’ or ‘pharmacy’ in their answers, the examples and context provided indicated that most respondents were predominantly answering from the lens of community pharmacy. Indeed, whilst mental health can be a specialty, it was acknowledged that mental health is relevant to pharmacists working in any sector.

*“I believe it is imperative that as pharmacists we are more aware of mental health—it is a sector we can work in but also we should be able to identify when patients are struggling.” (University 2, final year).*

#### Pharmacists are accessible

The nature of pharmacists, especially in the community, as the ‘first port of call’ was described. This encompasses practical accessibility including location and opening hours, frequency of contact and long-term relationships with patients, which support pharmacists to identify changes in mental health.

*“Pharmacy is often the first port of call for people who have a problem with their physical health and I feel that this could be extended into mental health as well. It's just as important to be able to offer the correct advice to someone for their mental health. I don't think that the information covered in the MPharm is enough to make me feel confident enough to deal with this scenario and therefore some extra training would be beneficial.” (University 25, final year).*

*“We are in a very privileged position to intervene as patients tend to visit community pharmacies more frequently than going to the GP. Pharmacists have a big role to play in mental health awareness and education.” (University 2, final year).*

#### Pharmacists could do more

Some respondents shared their vision for how the role of pharmacists in mental health care could be extended. This was particularly focused on community pharmacy roles and included raising public awareness of mental health and contribution to individual patient pathways through signposting and facilitating referral to resources and other healthcare professionals, including GPs and psychiatrists.

*“I still feel like we could do so much more to support people with mental health issues than simply counselling them on their medication. In particular, community pharmacists and primary care pharmacists are especially important as they have the time and the means to have conversations with people about their mental health more so than the increasing pressures placed on other sectors, e.g. hospital pharmacy teams, and have a opportunity to monitor and keep in contact with these patients.” (University 23, final year).*

*“Pharmacists have a unique opportunity to build a rapport with the patient and sign post them to other services, i.e. talking therapy and psychological interventions, to further boost their treatment outcome.” (University 25, final year).*

It was recognised that the current referral pathways might not be clearly defined or known by pharmacy teams.

*“We could do with more training on where to refer them within the community.” (University 23, final year).*

Finally, participants also identified a role for pharmacists in safeguarding the appropriate use of medicines, which they suggested was not always optimal in mental health.

*“Some initiatives to [be] made in community pharmacy to notify patients that they can get a consultation with mental health medication as there is over prescribing.” (University 22, year 3).*

#### Not a preferred role for everyone

Despite the overall impression of encouraging the pharmacist’s role, there were occasional outliers where respondents felt that mental health support is not within the community pharmacists’ remit.

*“I don’t think, as a community pharmacist, we would play more of a role in mental health. I am not sure where pharmacists working in different sectors (such as hospital or GP) would play more of a role in that, unless they’re based in a mental health hospital or ward. I always feel, perhaps wrongly I don’t know, that mental health problems are dealt with by the GP, specialist nurses and psychologists. Perhaps there is more of a role that pharmacists can play, but I feel that the current treatment and support that people with mental health problems receive is done by healthcare professionals other than pharmacists, unless directly relating to the drugs themselves.” (University 26, final year).*

One pharmacy student suggested that learning about mental health was not relevant to their future practice despite explicitly acknowledging that they would be supplying medicines for mental health.

*“I work in a community pharmacy and apart from seeing scripts for mental health drugs and handing out the patient’s medication I don’t see much to do with mental health.” (University 2, final year).*

### So, teach me

There was an overall impression that students wanted to understand more about mental health. They suggested ways in which this could be achieved. The first two subthemes highlighted a difference between those pharmacy students who acknowledged that they needed to learn more to increase competence which would be a necessity to them in future, and a smaller number who just seemed to want to learn for the value of the learning itself.

#### Wanting to learn

Data from a small number of respondents suggested that some pharmacy students just want to learn as much as they can, perhaps because they imagine that the subject will be interesting or alternatively through a recognition that whatever they learn is likely to have future value.

*“It is a subject I would love to understand a lot more about. I am unaware whether or not I will be taught it during the course but I hope I get the opportunity to do so.” (University 25, year 1).*

It was not possible here to determine what value participants ascribed to learning about mental health and as such these comments were categorised differently from those who felt that they *must* learn about mental health. Comments in which acknowledgement of immediate value was clearer were categorised accordingly (see below).

#### Needing to learn

Here, respondents identified a need to build confidence and communication skills related to mental health care and to be prepared to help people in mental health crises and linked this to a perceived necessity for more mental health education. Pharmacy students seemed to be imagining future professional scenarios that they felt ill-equipped to manage and were then linking this to something which might help.

*“Learning how to deal with the widest variety of people is very important to me; I want to be ready for any situation that may occur.” (University 25, year 1).*

Many respondents reported feeling underequipped and not confident to help people with their mental health. Some of this was attributed to limited focus on the mental health conditions and skills to support people with mental health in the MPharm.

*“it is a subject where most pharmacists/students have no more knowledge than the general public despite being the frontline of healthcare” (University 22, year 2)*

*“I guess there is more emphasis surrounding the drugs and how they work and dosages rather then discussing how to talk with someone about their mental health.” (University 18, final year).*

Much of the perceived lack of confidence was related to having supportive and effective conversations with people with mental health problems.

*“Learning and practising are completely different worlds, to help make us more confident speaking about mental [health] to people struggling with mental health, instead of awkward or thinking you have to walk on egg-shells with your words, undergoing training would make me personally feel more comfortable in treating and advising” (University 25, final year).*

*“If I’m counselling a patient who is very distressed, I feel completely under-equipped to be comforting and reassuring without making things worse. Particularly someone who has difficult behaviours such as delusions. I would just have no idea how to behave back. I'd probably just keep loudly repeating things.” (University 22, year 3).*

Responding to mental health crises was likely at the forefront of the respondents’ mind given the survey was centred around MHFA. Respondents described uncertainty of how to respond to mental health crises, much of which was inextricably linked to the described limited preparedness for effective communication.

*“I want to learn how to approach people with mental health on an emergency and especially when the patient has suicidal thoughts.” (University 23, final year).*

#### Gaps in training

The need for more focus on communicating about mental health problems and responding to crises, beyond pharmacology and physiology, has been described. Respondents additionally identified specific gaps in knowledge including eating disorders and personality disorders. One respondent identified under representation of mental health in the LGBTQ community.

*“Really wish they would delve more into LGBTQ community-specific issues, as the LGBTQ community is disproportionately affected by mental health illnesses.” (University 2, year 2).*

#### Learning from patients

More exposure to patients, specialty mental health settings and mental health pharmacists was identified by respondents as a potential way to improve competence. Pharmacy students suggested that they would value the opportunity to engage with patients and undertake more experiential learning.

*“I think more exposure would help, e.g. mental health nurses/students and placements with them, getting one to talk to us or show us effective mental health consultations. We did have a brilliant mental health pharmacist come speak to us about communication and it was really good, but maybe seeing a consultation would be even better.” (University 25, final year).*

*“In class teaching is one thing but to be prepared with dealing with any mental health cases, more placements/practical experience needs to be in place.” (University 25, year 3).*

#### Embedding throughout the curriculum

The respondents were across all levels of the MPharm. Those towards the ends of the degree reflected on mental health teaching across the MPharm and endorsed to the value of it being embedded throughout. The current picture suggests isolated modules, usually later in the degree. Students earlier in their studies seemed to assume that mental health teaching was to come.

*“I feel like it could be embedded throughout the programme rather than just focused on in third year and I’m assuming it’ll come up again in fourth year.” (University 2, year 3).*

*“Yet to study this topic but would be very interested. Also it would be good if it was embedded into the syllabus each year.” (University 25, year 1).*

#### Stigma

Stigma related to mental health was a theme which was found to crosscut the other main themes and sub themes. Some pharmacy students acknowledged the existence of stigma towards mental illness and suggested that pharmacists were well-placed to help counter this, particularly if they are equipped with the right tools in terms of prior learning.

*“We as pharmacists have a massive opportunity to change the way mental health has been perceived as we have greater contact with public.” (University 2, final year).*

*“I believe there is a lack of awareness around mental health issues. Education is an important step towards changing this.” (University 26, final year).*

Pharmacy students perceived these stigmatising attitudes to arise from a range of sources, including other students and lecturers, the general public and among healthcare professionals. Despite acknowledging this broad range of sources, students positioned themselves more positively, suggesting that they are less stigmatising than people around them:

*“There is so much misinformation and ignorance surrounding mental health that it will negatively impact the public’s health and wellbeing. If it is taught in a more empathetic way, and by less ignorant lecturers, it would make a huge difference. In addition, the students that make light of mental illness or refuse to acknowledge their mistakes were actually made to face consequences (at least a telling off and an explanation) it would benefit the health care system as a whole. We need to stop reinforcing the negative stigmas surround mental health.” (University 8, year 2).*

However, alongside describing the stigma that they perceived, and suggesting that pharmacists might be well-placed to counter it, some implicit stigma manifestations were apparent within the data. For example, discussion of how to ‘deal with’ patients. Pharmacy students seemed to endorse some mental illness stereotypes such as incompetence and unpredictability as well as subscribing to a them-and-us attitude.

## Discussion

Exploration of responses to the open-ended questions of the online survey provided insight into pharmacy students’ views on mental health teaching and learning and how this might impact on practice. Students articulated the importance of mental health in general, delineating the need to support mental health, illness, and wellbeing. They perceived that mental health difficulties had the potential to be overlooked. They recognised that mental and physical health were not separate entities and required parity as looking after all aspects of health was important for holistic care. Students highlighted the need to provide peer-support given concerns about anxiety and burnout in healthcare students.

### Interpretation

Students reflected on the role of the pharmacist, and this mainly appeared to be through a community pharmacy lens. They recognised that pharmacists have an important contribution to person-centred care. They identified that accessibility and frequency of contact with community pharmacists were key enablers. There was, however, an acknowledgement that pharmacists could do more for mental health care. Students highlighted that pharmacists were ideally placed to identify people with mental health difficulties and signpost to appropriate services, particularly if they were trained and supported to do so. In a recent study of pharmacists in New Zealand, participants were more interested, but less confident, in providing mental health care than care for cardiovascular disease [21]. This may be a marker of growing interest and responsibility relating to mental health, although responses may have been influenced by social desirability, as described by study authors.

Some students did refer to the role of the pharmacist from a specialist mental health perspective, but this was limited. This is in contrast to increasing recognition for the role of the specialist mental health pharmacist by team members and patients for providing comprehensive medicines management services [22]. There were no comments about role of hospital pharmacists in general hospitals in relation to mental health care, and there is a paucity of evidence in the literature about this role.

Some students seemed uncertain of the role of pharmacists in mental health and interestingly, failed to identify that dispensing medicines for mental health difficulties provided opportunities for conversations and care. A prescription is often the prompt for enrolment in community pharmacy mental health services, but in both the Bloom and AMPLIPHY programs, patient-led agendas were often wider than medication-related issues [10, 12].

Students highlighted the need for teaching and learning in the MPharm to incorporate communication, confidence in responding to mental health needs and especially in crisis. This corroborates quantitative findings from the same questionnaire, [16] where less than half of student (45%) stated they practised counselling about mental health medication. There seems little progress since Rutter et al.’s findings a decade ago [15] which expressed the need for more confidence and competence in communication and problem-solving about mental health. Participants identified learning from patients to improve confidence and communication. People with lived experience of mental health have contributed to teaching and assessing students [23]. Examples of where people with lived experience have enhanced mental health provision in pharmacy degrees include via patient and public involvement in teaching, [24, 25] placement, [26] in curriculum design [19, 27, 28] and as simulated patients to assess skills [19].

MHFA training is one approach to improve students’ confidence and competence in supporting mental health crises that has been adopted in pockets of pharmacy schools [17, 19, 29, 30]. Other pharmacy schools have included bespoke suicide prevention training [31–33]. However, to our knowledge, there is no uniform approach across individual countries, nor one mandated by the respective pharmacy regulators. There is evidence this type of training would be welcomed by pharmacy students [16].

There is evidence in the literature that there is substantial internal stigma in pharmacy students, yet low rates of stigma towards others [34]. In a survey of 256 pharmacy and pharmaceutical science students at a USA university, 11% reported stigma-related barriers to help-seeking for mental health and 63% reported internal stigma [35]. In the quantitative findings for this present study, 81% of students agreed that stigma relating to mental health exists [16]. Of particular concern, is the observation from a study of pharmacy student attitudes in Estonia, which found that students were unlikely to associate negative attitudes with barriers to patient care [36].

### Strengths

This questionnaire obtained views from pharmacy students from 18 universities across UK and Ireland [16]. It generated many qualitative comments about mental health in the curricula, enabling an in-depth exploration, for the first time since 2013 [15].

### Limitations

These findings must be interpreted with awareness of the lens from which they were generated. The preceding questions had directed students to think about MHFA, which might have drawn out comments to do with crises and comparisons with physical health. Respondents self-selected to participate in this study. This might have attracted those at the extremes of views, especially passionate or, conversely, disillusioned about the mental health role of pharmacists. They may not represent the views of students at all pharmacy schools in the UK and Ireland. This might explain some of the contrasts highlighted in the above themes.

## Conclusion

Pharmacy students appreciated the importance of mental health care and promoting mental health and wellbeing. They expressed the view that mental health was under-recognised and needed parity with physical health. Most pharmacy students articulated the value of the role of the pharmacist in providing person-centred care. While noting mental health to be important in other sectors, respondents placed a particular emphasis on the role of the community pharmacist. Accessibility of community pharmacy was seen as an enabler to further develop the role; improving signposting for those with mental health difficulties and medicines optimisation. Students, therefore, wanted to learn more about mental health and identified that they needed to learn more to have the skills and confidence to help people in difficulty. They suggested strategies to improve learning, including integrating mental health throughout the curriculum and having more opportunities to learn from patients. Stigma pervaded all themes, with students acknowledging stigma in healthcare and reporting stigma in education. Pharmacy students are willing and will be better able to provide person-centred mental health care when we equip them to do so.
